# Cord blood DNA methylation reflects cord blood C-reactive protein levels but not maternal levels: a longitudinal study and meta-analysis

**DOI:** 10.1186/s13148-020-00852-2

**Published:** 2020-04-30

**Authors:** Edwina H. Yeung, Weihua Guan, Xuehuo Zeng, Lucas A. Salas, Sunni L. Mumford, Paula de Prado Bert, Evelien R. van Meel, Anni Malmberg, Jordi Sunyer, Liesbeth Duijts, Janine F. Felix, Darina Czamara, Esa Hämäläinen, Elisabeth B. Binder, Katri Räikkönen, Jari Lahti, Stephanie J. London, Robert M. Silver, Enrique F. Schisterman

**Affiliations:** 1 Epidemiology Branch, Division of Intramural Population Health Research, Eunice Kennedy Shriver National Institute of Child Health and Human Development, 6710B Rockledge Dr, MSC 7004, Bethesda, MD 20817 USA; 2 Division of Biostatistics, School of Public Health, University of Minnesota, A460 Mayo Building, MMC 303, 420 Delaware St. SE, Minneapolis, MN 55455 USA; 3 Glotech, Inc., Bethesda, MD 20817 USA; 4 Department of Epidemiology, Geisel School of Medicine at Dartmouth College, Lebanon, NH 03766 USA; 5 ISGlobal, 08003 Barcelona, Spain; 6 Universitat Pompeu Fabra (UPF), 08003 Barcelona, Spain; 7 CIBER Epidemiología y Salud Pública (CIBERESP), Madrid, Spain; 8 The Generation R Study Group, Erasmus MC, University Medical Center Rotterdam, P.O. Box 2040, 3000 CA Rotterdam, The Netherlands; 9 Department of Pediatrics, Erasmus MC, University Medical Center Rotterdam, P.O. Box 2040, 3000 CA Rotterdam, The Netherlands; 10 Department of Psychology and Logopedics, Faculty of Medicine, University of Helsinki, Helsinki, Finland; 11 IMIM (Hospital del Mar Medical Research Institute), 08003 Barcelona, Spain; 12 Department of Translational Research in Psychiatry, Max-Planck-Institute of Psychiatry, Munich, Germany; 13 University of Eastern Finland, Kuopio, Finland; 14 Department of Psychiatry and Behavioral Sciences, Emory University School of Medicine, Atlanta, USA; 15 Division of Intramural Research, National Institute of Environmental Health Sciences, National Institutes of Health, Department of Health and Human Services, Research Triangle Park, Durham, NC 27709 USA; 16 University of Utah, Salt Lake City, 50 N Medical Dr, Salt Lake City, UT 84132 USA

**Keywords:** Inflammation, C-reactive protein, DNA methylation, Pregnancy, Newborn, Developmental programming

## Abstract

**Background:**

Prenatal inflammation has been proposed as an important mediating factor in several adverse pregnancy outcomes. C-reactive protein (CRP) is an inflammatory cytokine easily measured in blood. It has clinical value due to its reliability as a biomarker for systemic inflammation and can indicate cellular injury and disease severity. Elevated levels of CRP in adulthood are associated with alterations in DNA methylation. However, no studies have prospectively investigated the relationship between maternal CRP levels and newborn DNA methylation measured by microarray in cord blood with reasonable epigenome-wide coverage. Importantly, the timing of inflammation exposure during pregnancy may also result in different effects. Thus, our objective was to evaluate this prospective association of CRP levels measured during multiple periods of pregnancy and in cord blood at delivery which was available in one cohort (i.e., Effects of Aspirin in Gestation and Reproduction trial), and also to conduct a meta-analysis with available data at one point in pregnancy from three other cohorts from the Pregnancy And Childhood Epigenetics consortium (PACE). Secondarily, the impact of maternal randomization to low dose aspirin prior to pregnancy on methylation was assessed.

**Results:**

Maternal CRP levels were not associated with newborn DNA methylation regardless of gestational age of measurement (i.e., CRP at approximately 8, 20, and 36 weeks among 358 newborns in EAGeR). There also was no association in the meta-analyses (all *p* > 0.5) with a larger sample size (*n* = 1603) from all participating PACE cohorts with available CRP data from first trimester (< 18 weeks gestation). Randomization to aspirin was not associated with DNA methylation. On the other hand, newborn CRP levels were significantly associated with DNA methylation in the EAGeR trial, with 33 CpGs identified (FDR corrected *p* < 0.05) when both CRP and methylation were measured at the same time point in cord blood. The top 7 CpGs most strongly associated with CRP resided in inflammation and vascular-related genes.

**Conclusions:**

Maternal CRP levels measured during each trimester were not associated with cord blood DNA methylation. Rather, DNA methylation was associated with CRP levels measured in cord blood, particularly in gene regions predominately associated with angiogenic and inflammatory pathways.

**Trial registration:**

Clinicaltrials.gov, NCT00467363, Registered April 30, 2007, http://www.clinicaltrials.gov/ct2/show/NCT00467363

## Background

Inflammation is a non-specific response to insults to the body. A five-fold increase in inflammation, as measured by CRP levels, occurs over the course of normal pregnancy [1], and the fetal impact is unknown. Elevated inflammation beyond the physiological increase during pregnancy has been implicated as a mediator in the associations of a myriad of prenatal exposures (e.g., infectious disease [2], maternal obesity [3, 4], maternal stress [5], and air pollution [6]) with offspring health. Moreover, maternal inflammation is linked with preterm birth [7, 8], preeclampsia [9, 10], and childhood asthma/allergies [11–14]. Also, women with chronic inflammatory diseases such as rheumatoid arthritis have lower weight babies [15]. Developmental programming studies suggest that prenatal exposures could alter long-term offspring health through epigenetic changes, often effected via DNA methylation. DNA methylation is one epigenetic modification that has become frequently studied due to the availability of reliable and affordable technologies.

C-reactive protein (CRP) is a cytokine routinely used to measure inflammation. CRP is an acute phase reactant produced by the liver, and serum levels can increase 3000-fold within a short amount of time as part of innate immunity [16]. Its plasma half-life is about 18 h but in the acute phase may only be 75 min [16]. Rapid increase in CRP is associated with infections rather than prenatal exposures or systemic inflammation. While many cytokines including interleukins and interferons are part of the inflammatory response, CRP is most often studied due to reliable assay and clinical use as a global marker of systemic chronic inflammation. In adults, circulating CRP has been cross-sectionally associated with altered DNA methylation patterns in white blood cells in a meta-analysis of epigenome-wide studies with data from 8863 Europeans and 4111 African Americans [17]. Moreover, evidence from a longitudinal study of over 100 adults shows DNA methylation changes specific to CRP levels measured 10 years prior, suggesting lasting influence of inflammation on DNA methylation [18]. However, it is unclear whether CRP throughout pregnancy has similar effects on newborn methylation, and whether the timing of the exposure at different gestational ages, including at delivery, matters. Longitudinal analyses of CRP during pregnancy, therefore, could be informative in understanding the role of inflammation on newborn methylation. Decreasing maternal inflammation could also be another way to demonstrate a causal role, and low dose aspirin use may decrease maternal CRP [19], which may in turn alter newborn methylation.

We aimed to comprehensively identify newborn DNA methylation differences associated with prenatal CRP exposure. In the Effects of Aspirin in Gestation and Reproduction (EAGeR) study, high-sensitivity CRP levels were measured longitudinally in maternal serum at three time points during pregnancy and in cord blood at delivery. Newborn DNA methylation was assessed in cord blood obtained at delivery. In addition, as part of the Pregnancy And Childhood Epigenetic (PACE) consortium [20], maternal CRP levels measured in the first trimester and DNA methylation in cord blood were available from three international pregnancy cohorts.

## Results

Women in EAGeR had an average pre-pregnancy BMI of 25.2 kg/m^2^, predominantly did not smoke, and 43% had household income levels above $100,000 (Table 1). Levels of maternal and cord blood CRP are provided in Table 2. No significant associations between maternal CRP concentrations during pregnancy were found regardless of gestational age at measure in EAGeR (i.e., in first, second, or third trimester) in crude models adjusted for covariates (i.e., Model 1: maternal age, smoking, income, pre-pregnancy BMI, plate) or in two models additionally adjusting for cell type distribution using two different cord blood reference panels (i.e., Model 2, Bakulski [21]; Model 3, Gervin [22] (Supplemental Tables A–C in the online repository, Figshare)). In sensitivity analysis, we repeated all analyses without exclusion of CRP >10 mg/dl so as to not exclude acute infections but found no associations aside from one CpG with third trimester CRP levels which was not in a known gene region (cg14503868; beta = − 0.02, FDR *p* = 0.01; located in Chr 9, position 128504605, closest to *PBX3*) (See Supplemental Tables A4, B4, C4 on Figshare).

**Table 1:** EAGeR maternal and infant characteristics

			Placebo		Low-dose aspirin		
*n* = 358	*n*/mean	%/SD	*n*/mean	%/SD	*n*/mean	%/SD	*p* value
Maternal age (years)	28.2	4.4	28.2	4.6	28.2	4.3	0.91
High school or more	320	89%	157	91%	163	88%	0.27
Randomized to aspirin	185	52%	0	0	185	100%	n/a
Smoking	10	2.8	4	2.3	6	3.2	0.30
≥ $100,000 income	152	43%	68	39%	84	45%	0.65
Married	346	97%	163	94%	183	99%	0.03
BMI (kg/m^2^)	25.2	5.6	25.8	6.0	24.6	5.1	0.05
Female baby	179	50%	93	54%	86	46%	0.20
Gestational age (wk)	38.9	1.6	38.9	1.73	38.9	1.37	0.98
Birth weight (g)	3347.0	475.0	3358.3	514.5	3336.6	436.2	0.67

Missing (*n*), BMI (3), smoking (2)

**Table 2:** Distribution of CRP concentrations

Study	Timing of measure	*n*	GA (week) of measure	Mean CRP	SD CRP	Median CRP	CRP range (min–max)
EAGeR	Preconception	350	n/a	1.7	1.8	0.9	0.11–9.96
EAGeR	1st trimester	322	8.8	3.0	2.4	2.3	0.15–9.86
EAGeR	2nd trimester	304	20.9	4.2	2.4	3.7	0.15–10
EAGeR	3rd trimester	320	36.5	3.8	2.2	3.4	0.17–9.94
EAGeR	AUC log (CRP)	359	n/a	46.2	14.4	43.8	13.98–97.83
EAGeR	Delivery CRP	358	Delivery	0.2	0.4	0.1	0–5.67
INMA	1st trimester	266	13.5	4.4	2.1	4.0	2.00–9.38
PREDO	1st trimester	242	13.0	3.7	2.4	3.1	0.23–9.78
Generation R	1st trimester	773	13.1	3.9	2.5	3.3	0.3–10.0

Similarly, in the meta-analysis with three PACE cohorts for first trimester CRP measures, no significant associations were found. Table 3 lists the top 5 associations (all FDR *p* > 0.05) in the meta-analysis and the direction of association from each cohort. Supplemental Table 1 provides characteristics of the participants in the other cohorts, and Supplemental Table 2 provides information on the genomic inflation of the various models run from each study.

**Table 3:** Meta-analysis of first trimester CRP and newborn methylation in four cohorts

CpG Site	Effect	SE	*p* value*	Direction**	Het. *p* value	Chr: position
cg19858671	0.0041	8.00E−04	1.21E-06	++?+	0.83	8: 130951373
cg06320401	− 0.0092	0.0019	1.50E-06	----	0.74	1: 48176755
cg22649187	− 0.0069	0.0015	3.56E-06	----	0.88	11: 48176755
cg04747517	− 0.0022	5.00E−04	3.89E-06	----	0.73	3: 169755595
cg05986933	− 0.0042	9.00E−04	4.11E-06	----	0.84	2: 43020457

*SE* standard error, *Het* heterogeneity, *chr* chromosome

*All FDR *p* values < 0.5 (non-significant) and were adjusted for maternal age, race (as applicable), socioeconomic income (by education, income, or other cohort specific factors), maternal BMI and smoking, and cell type distribution [21] (Model 2)

**Direction of associations for each cohort in the following order: GenR, PREDO, INMA, EAGeR; the “?” is due this CpG being excluded from analysis in INMA after data cleaning steps

One association with newborn methylation was identified in additional analyses performed in EAGeR to evaluate whether cumulative inflammation over pregnancy (as estimated by the area under the curve of CRP measured in gestational weeks 8–36) was associated with DNA methylation in offspring (Supplemental Tables D1-3 on Figshare). The CpG (cg09180262) was significant only for Model 3 with small effect size (− 0.0002 per log unit CRP). We also assessed “the extremes” by comparing newborn methylation in infants born to women consistently in the highest tertile of CRP (*n* = 64) for all 3 trimesters of pregnancy compared to newborns of women consistently at the lowest tertile (*n* = 62). In this analysis, DNA methylation levels at 5 CpGs were associated with CRP, the most highly associated CpG being in the intron of the major histocompatibility complex, class II, DQ beta 1 (*HLA-DQB1*). See Supplemental Table 3 for annotated results of the top 6 CpGs and Supplemental Tables D–E on Figshare for full results.

In the analysis comparing newborn CRP measured in cord blood at the same time as DNA methylation, 108 significant associations were observed in crude models (Model 1) without cell-type adjustment initially in EAGeR. After Model 2 adjustment using the Bakulski reference [21] to estimate cell type distribution, 71 significant associations were found. This number was further reduced in Model 3 after adjustment for cell type as estimated by Gervin et al. [22], and 33 significant associations were identified (Table 4). Supplemental Figure 2 shows the overlap in the associations found between the 3 models. Of all the CpGs identified, 20 were FDR-significant in all three models regardless of cell type adjustment, whereas 6 associations (as marked by asterisks in Table 4) were significant only in Model 3. Effect sizes of Model 3 associations ranged from 2 to 15% differences in methylation per log unit change in CRP at delivery, and the top 8 CpGs were Bonferroni significant (*p* < 6 × 10^−8^). There were almost equal numbers of CpGs that had higher methylation (18 CpGs) as lower methylation (15 CpGs) in relation to increasing concentrations of CRP at delivery. The top CpG (cg13138089, *p* = 2 × 10^−14^) is in the CpG island for the gene *ECEL1P2* (Endothelin Converting Enzyme Like 1 Pseudogene 2), which is relevant for endothelial function. Apart from the 3 CpGs on the X chromosome, 3 associated with non-coding RNAs, and 4 with no known genes within 5 kb, the gene locations and functions of the remaining 23 CpGs were reviewed (Supplemental Text). Among the 33 significant CpGs, no significant cell type specific signals were identified by eFORGE. However, enrichment for transcription factors related to immune cell lineages preserved across evolutionary lines was noted from several databases (Supplemental Figure 3 and Supplemental Table 4).

**Table 4:** Delivery CRP and newborn DNA methylation in EAGeR

CpG	Beta	SE	*p* value	FDR *p* value	chr	pos	Gene name	Relation to island**
cg13138089	0.152	0.01988	2.11E−14	1.73E−08	2	233251770	*ECEL1P2*	Island
cg23289135	− 0.1253	0.01731	4.63E−13	1.90E−07	17	6182962		OpenSea
cg02323356	0.05283	0.008898	2.89E−09	0.00065	2	220313153	*SPEG*	Island
cg18875674	− 0.07196	0.01215	3.19E−09	0.00065	11	73026651	*ARHGEF17*	OpenSea
cg13558754	0.09236	0.01572	4.22E−09	0.00069	19	36247867	*HSPB6; PROSER3*	Island
cg16426346	− 0.09836	0.01706	8.14E−09	0.0011	6	1377047		N_Shore
cg20789824	− 0.02913	0.005279	3.42E−08	0.004	9	127562861	*OLFML2A*	OpenSea
cg17990365	− 0.04933	0.009036	4.79E−08	0.0049	11	319718	*IFITM3*	S_Shore
cg10884341	0.02292	0.004233	6.16E−08	0.0056	2	198365065	*HSPE1*	Island
cg20264732	0.02438	0.004544	8.05E−08	0.0066	16	68269763	*ESRP2*	Island
cg19013417	− 0.02286	0.004313	1.17E−07	0.0087	X	6146809	*NLGN4X*	S_Shore
cg19922333	− 0.04085	0.007806	1.66E−07	0.011	2	218253142	*DIRC3*	OpenSea
cg24340661	− 0.02255	0.004367	2.43E−07	0.012	1	166944531	*MAEL; ILDR2*	N_Shore
cg17238334	− 0.03159	0.00611	2.33E−07	0.012	5	71920983	LINC02056	OpenSea
cg23200634	− 0.09106	0.01763	2.39E−07	0.012	11	68709832	*IGHMBP2*	OpenSea
cg04415152*	0.03967	0.00764	2.08E−07	0.012	11	119383197	USP2-AS1	OpenSea
cg12615852	− 0.06756	0.01297	1.92E−07	0.0126	14	106330121		N_Shelf
cg12499872	0.06191	0.0121	3.09E−07	0.014	16	58019893	*TEPP*	Island
cg02660803	− 0.09608	0.01885	3.44E−07	0.015	X	91033070	*PCDH11X*	N_Shore
cg23133011	0.03135	0.006176	3.84E−07	0.016	11	122847242	*BSX*	N_Shore
cg25460807	0.04084	0.008097	4.55E−07	0.018	8	21908022	*DMTN*	S_Shelf
cg18449964	0.03482	0.006925	4.94E−07	0.018	18	72917101	*ZADH2*	Island
cg25389127	− 0.01482	0.002998	7.62E−07	0.027	X	8700496	ANOS1	Island
cg22876643	0.03535	0.007197	9.06E−07	0.03	1	68962318	*DEPDC1*	Island
cg04136921	− 0.05492	0.01121	9.67E−07	0.03	11	5626314	*TRIM6; TRIM6-TRIM34*	OpenSea
cg26354128	0.0325	0.006636	9.72E−07	0.03	14	93897195	*UNC79*	Island
cg19766763*	0.0307	0.006247	8.93E−07	0.03	18	61604237	*SERPINB10*	Island
cg19561181*	− 0.05247	0.0108	1.17E−06	0.03	20	56794558	*ANKRD60*	OpenSea
cg10235275*	0.03552	0.007398	1.58E−06	0.04	10	65225544	*JMJD1C*	Island
cg09689461*	0.0601	0.0126	1.84E−06	0.048	1	163130728	*LOC101928404; RGS5*	OpenSea
cg03305585	0.0339	0.007105	1.83E−06	0.048	7	37805796		OpenSea
cg21542650*	0.03161	0.00663	1.87E−06	0.048	12	2903593	*FKBP4*	Island
cg18973939	0.03444	0.007235	1.94E−06	0.048204347	1	16164122	*FLJ37453*	Island

Model 3 adjusted for maternal age, smoking status, income, pre-pregnancy BMI, plate, and cell count distribution (as estimated in Gervin et al. [22])

Asterisk (*) indicates CpG not previously identified in previous crude models or model 1 adjusting for cell type using Balkulski et al. [21]

Double asterisks (**) as defined by Illumina [23]

Lastly, approximately half of the EAGeR participants were randomized to low-dose aspirin as expected (Table 1). Randomization to low dose aspirin was not associated with differences in newborn methylation (Supplemental Figure 1), except for DNA methylation at one CpG (cg2002882, chromosome 13, position 79169823, beta = 0.009, FDR-*p* = 0.04).

## Discussion

We report, to our knowledge, the first study to examine the longitudinal association between prenatal inflammation as repeatedly measured by CRP and newborn DNA methylation at delivery (Supplemental Figure 4). Associations relevant to inflammation/angiogenesis were identified in newborns at delivery suggesting that CRP, as an acute phase reactant, associates with methylation of circulating cells when measured at the same time point, reiterating CRP’s role in inflammatory and angiogenic pathways although the temporality of these associations remains unclear. Few associations in genes related to histocompatibility proteins/antigens were identified with persistent high CRP.

### CRP during pregnancy

The general lack of association between circulating maternal CRP levels during pregnancy and newborn DNA methylation may be related to several reasons. First, due to its short half-life, CRP may represent transient acute inflammation rather than chronic inflammation. However, few associations emerged even when we modeled longitudinal CRP levels in two different ways to capture women with relatively and chronically higher levels throughout pregnancy. These analyses were done by modeling cumulative CRP using an area under the curve model with repeated measures and by categorizing women consistently in the top tertile of CRP from first through third trimester. For the latter, we observed a few differences in DNA methylation located at genes related to immune function after cell type adjustment. *HLA-DQB1* codes for a class II major histocompatibility complex protein, *PUM3* codes for a minor histocompatibility antigen, and *MYO1G* codes for a minor histocompatibility antigen precursor. In other studies evaluating prenatal exposures on offspring methylation, *HLA-DQB1* methylation differed between siblings at 4–6 years of age before and after maternal weight loss from bariatric surgery [24], and *MYO1G* methylation was associated with environmental chemical exposure [25]. However, these findings require replication given the selectiveness of the sample.

As CRP does not cross the placental barrier [26], its indirect influence on neonatal health may be modified by downstream factors including immune responses. And while the majority of previous studies have found maternal CRP to be associated with smaller newborn size [27, 28], it is inconsistently associated with other conditions [2, 29]. Studies linking maternal CRP with childhood asthma/allergies [12, 14] have also been inconsistent. These observations, in combination with the natural rise of CRP during pregnancy [30], make it difficult to evaluate normative rather than “elevated” CRP in the context of pregnancy. We modeled linear associations with the understanding that higher inflammation is indicative of cellular damage, but in the context of pregnancy, it is possible that some non-linear relationships exist for the lowest and the highest CRP levels. Second, CRP levels have been shown not to correlate with other cytokines measured early in pregnancy [31]. Thus, other biomarkers that may provide a fuller picture of prenatal inflammation may be needed to further investigate the role of inflammation. Third, cord blood CRP levels were 10- to 20-fold lower than maternal CRP levels (Table 2), despite evidence that maternal CRP increases dramatically during labor and delivery [32]. Placental production of CRP is largely directed into maternal circulation [33]. Thus, natural protective mechanisms may cause maternal CRP to not reflect local inflammation in the developing fetus and explains why it is not associated with newborn methylation. Last, the sample size of EAGeR (*n* = 391) has limited power to detect differences. Nevertheless, a meta-analysis of over 1600 newborns and the findings for CRP at delivery in EAGeR suggest otherwise. Taken together, maternal CRP levels during pregnancy are generally not associated with alterations in DNA methylation patterns to the offspring as measured at delivery in cord blood.

Randomization to low-dose aspirin was also largely not associated with differences in newborn methylation. The single association identified on chromosome 13 (cg20028827) after accounting for multiple testing (FDR *p* = 0.04) was located ~ 3.5 kb upstream of the promoter of *POU4F1* (chr13:79173227-79177695). It is in a DNase hypersensitive site (chr13:79169325-79170815) with peaks for transcription factors EZH2 and SUZ12, among others. The levels of H2K4Me1 are slightly higher but with no apparent enhancer overlap. However, the CpG is within the intron of several ncRNA variants of the OBI1-AS1 (previously known as the PUF1-AS1 or RNF219-AS1). A log unit increase in CRP levels was associated with a < 1% increase in methylation levels (adjusted beta = 0.009). *POU4F1* codes for neural transcriptors, particular to ganglions, by playing a role in development of calcium channels [34]. However, its methylation has only been examined with regard to breast tissue tumors (where it was found to be hypermethylated and its expression silenced) [35].

### CRP at delivery

While no associations with maternal CRP were identified, newborn CRP levels were associated with differences in cord blood methylation. Interestingly, 13 of the CpGs were located in or near genes associated with inflammatory or angiogenic pathways (*ECEL1P2*, *SPEG*, *ARHGEF17*, *OLFML2A*, *HSPB6*, *HSPE1*, *IFITM3*, *ZADH2*, *DEPDC1*, *TRIM6*, *SERPINB10*, *JMJD1C*, and *RGS5*). Of note, the top 7 genes, 5 of which exceeded Bonferroni significance, are among these genes. eFORGE results also confirm these methylation changes corresponded to transcription factors involved in development of immune cell lineages. These observations further support their biological relevance in relation to CRP. Previous studies that have measured inflammation and methylation in adults have been systematically reviewed [36]. We compared the cross-sectional associations found in the current analysis at birth with those of previous epigenome-wide association studies among adults but were unable to find overlap between statistically significant CpGs identified by other studies and our results [17, 18]. All but one study used the 450K panel, such that most of the identified CpGs in the current analysis are unique. We add to the list of CpGs identified, which include sites that are novel to the EPIC microarray. Myte et al. also used the EPIC microarray but had a limited sample size of < 200 adults [18]. Cord blood samples unlike those from older children or adults normally contain nucleated red blood cells, which may also explain differences among studies [37]. Moreover, immune system development continues after delivery as the newborn comes into contact with microbes, whereas adult immune systems have matured with respect to a range of environmental exposures and their own microbiome [38].

### Limitations

EAGeR included women with 1–2 previous pregnancy losses. We cannot exclude that genetic causes of pregnancy loss [39], including a role for CRP [40], may play a role. This may limit the generalizability of the EAGeR results. However, the PACE birth cohorts contributing to the first trimester analysis were recruited from the general population and echoed the same findings with regards to maternal CRP. The small sample size of EAGeR and the meta-analysis may have limited power to detect weaker associations. Given that inflammation plays an integral role in recruitment of white blood cells, using a mix of cells as in buffy coat or any blood sample without prior selection of cell type for DNA methylation measurement requires estimation of cell type based on previous reference panels [21, 22]. Remaining estimation errors cannot be excluded. Also, the specificity of the associations identified to CRP is unclear. Our results are based on cord blood derived methylation and unclear if generalizable to other tissues. CRP is just one acute phase protein (APP), and various cytokines/chemokines either leading to APP production are increased by APPs (e.g., interleukins, TNF-a) in turn recruits immune cells. Thus, associations might have been due to correlated cytokine production and upstream factors. Lastly, the Illumina platform covers a large number of CpG sites but only a small fraction of the genome. It might not provide sufficient coverage to evaluate differences in global methylation as other techniques [41]. Nevertheless, site specific differences identified in this approach revealed specific genes and biological pathways related to inflammation.

### Conclusion

In conclusion, DNA methylation levels as measured in cord blood do not correlate with prenatal levels of CRP over pregnancy. Rather, methylation levels are consistent with concurrently measured CRP. The differentially methylated CpG sites suggest pathways that are modulated by inflammatory cytokines. Future research in larger studies and using different biomarkers of inflammation apart from CRP are needed for understanding the role of prenatal inflammation in fetal and neonatal developmental programming. Other epigenetic mechanisms such as histone acetylation and non-coding RNAs may also provide insights into the role of prenatal inflammation.

## Methods

### Study design

The Effects of Aspirin in Gestation and Reproduction (EAGeR) trial (2007–2011; NCT00467363) randomized women prior to conception who previously experienced 1–2 prior pregnancy losses to 81 mg low-dose aspirin and 400 μg folic acid, or placebo plus 400 μg folic acid [42]. Randomization to low-dose aspirin did not significantly alter live birth rates [42]. Its design and results are detailed elsewhere [42]. The current analysis was nested among 391 newborns with DNA methylation data available from the Utah site [43]. The University of Utah IRB approved the study (Salt Lake City, Utah IRB #1002521), and all participants provided written informed consent prior to enrollment. Among the 391 newborns with methylation data, we excluded 12 newborns of non-white self-reported ethnicity (to avoid population stratification in meta-analysis across cohorts) and 9 for missing CRP measures.

### Laboratory methods

Women in EAGeR underwent multiple blood draws; first at baseline prior to pregnancy and at approximately 8, 20, and 36 weeks gestation during pregnancy. In addition, among 428 deliveries from the Utah site, 10 mL cord blood was collected in a plasma collection tube with ethylenediaminetetraacetic acid (EDTA) [43]. CRP was quantified from maternal serum samples and cord blood plasma by means of an immunoturbidimetric assay (Roche COBAS 6000, Roche Diagnostics, IN) with a limit of detection (LOD) of 0.15 mg/L. Cord blood DNA underwent bisulphite conversion with standardized kits (e.g. Zymo EZ DNA MethylationTM kit, Zymo, Irvine, CA), followed by measurements of DNA methylation using the Infinium MethylationEPIC BeadChip microarray [43].

## Statistical analysis

### Data cleaning for EAGeR

Methylation data were processed using the minfi package in R [44], including the identification of failed probes and scaling with Illumina control probes to determine methylation values. The beta value (*β*) was determined for each of the CpG sites by the fluorescent signals (*β* = Max (*M*, 0) / [Max (*M*, 0) + Max (*U*, 0) + 100), where *M* and *U* are the intensity of methylated and unmethylated allele [45]. *β* values approaching 1 are completely methylated, and those close to 0 are unmethylated. Background and dye-bias corrections were applied. Quantile normalization was used to normalize beta values between two types of probes [46]. The purpose of this step is to eliminate potential probe type bias (type I vs II probes). Cell type mixture was then estimated on the full set of normalized data (FlowSorted.CordBlood.450K package) [21]. Principal component analysis (PCA) was performed to further detect outliers and samples mismatched in sex compared against information from electronic medical records. Five samples with sex mismatch were further excluded. We extracted the detection *p* value for each methylation measure (per site per sample) and filtered data that failed detection *p* value (*p* > 0.01). Beta values were replaced as missing if either detection *p* value > 0.01 or bead counts < 3. We removed samples and CpG sites with low passing rate (< 97%) based on detection *p* value and bead counts. After probe removal, 821,665 CpG probes remained.

### Modeling exposure for EAGeR

To exclude acute inflammation from infection, maternal and cord plasma samples with CRP above 10 mg/L were excluded from analysis (15 at preconception, 39 at 8 weeks, 54 at 20 weeks, 28 at 36 weeks, 1 at delivery from EAGeR). Among the 391 neonates with DNA methylation data, 358 remained for analyses with CRP measured at any time during pregnancy. CRP was natural log transformed for normality. Longitudinal measures were also used in combination to examine inflammation in two ways. First, the cumulative concentration of CRP (log transformed) across the first, second, and third trimesters was approximated using linear mixed models. The linear mixed model was fitted with a random intercept and random centered week of CRP measurements. The model estimates also incorporated fixed effects including an intercept and a linear and quadratic term of centered week of CRP measurements, based on the regression curve of the original CRP concentrations. For each subject, parameters from the model were used to define the predicted CRP curves. The area below the curves was divided into 1000 parts. The area of each part was then calculated as a rectangle, since they are very small, and summed to obtain the cumulative CRP concentrations over pregnancy [47]. In addition, methylation was also examined by comparing two extreme groups of women that is women in the top tertile of CRP across all trimesters were compared with women in the bottom tertile across all trimesters. Cumulative and tertile analyses did not exclude maternal CRP levels above 10 mg/L to better characterize inflammation across the whole of pregnancy for the same woman. While the primary analysis excluded CRP levels above 10 to rule out outliers that would have strong influence on estimates based on linear regression models, it would have excluded too many women for cumulative or tertile analyses who just had one of the 3 with an “elevated” measure. The cumulative and tertile analyses were more robust to outlying CRP.

### Modeling outcomes for EAGeR

Trimming of the outlying methylation values at IQR ± 3 × IQR (IQR = inter-quartile range) was performed after values were normalized. Complete case analyses were performed without imputation. Linear mixed effects models were used to test associations between methylation beta values at each CpG site and measured CRP at each time point and as cumulative or by extreme tertiles with adjustment for covariates. Batch effects (as covariates of chip and row) were accounted for through random effects. FDR-correction was applied to account for multiple testing.

### Covariates for EAGeR

Covariates included maternal age (continuous, by self-report), smoking (yes/no by self-report), income (5 categories from ≥ $100,000, $75,000–99,999, $40,000–69,999, $20,000–19,999, ≤ $19,999 by self-report), pre-pregnancy BMI (continuous, by direct measure prior to pregnancy), plate (for batch effects), and with or without cell count distribution (in separate models). Rather than adjustment for self-reported ethnicity, 12 non-white participants were excluded from EAGeR analyses. Three models were run; Model 1 adjusting for covariates and two models additionally adjusting for cell type distribution using two separate reference panels. Model 2 adjusted for covariates and cell counts based on a cord blood reference using *minfi* in R [21], including B-cell, CD-4+ T cells, CD-8+ T cells, granulocytes, monocytes, NK-cells, and nucleated RBCs. A major difference previously identified between adult and cord blood cell count distribution in terms of DNA contribution from buffy coat samples is the proportion of nucleated red blood cells [21]. Model 3 used a more recent cord blood reference pooling together larger number of samples with available cell distribution information [22].

### Meta-analysis

As part of the PACE consortium [20], three pregnancy cohorts (INMA [48], Generation R [49], PREDO [50]) provided results for the associations between CRP measured in first trimester samples and DNA methylation measured in cord blood at delivery adjusting for the covariates mentioned above including with and without adjustment for cell type using two different references as described above [21, 22]. A meta-analysis of results was conducted with EAGeR as the fourth cohort. Inverse-variance weighted fixed effects meta-analysis was performed among a total of 1603 newborns using the software METAL [51]. Specific analytical and laboratory methods are included in Supplemental materials for each of the cohorts.

### Bioinformatics

All FDR-significant CpGs were searched in the UCSC Genome Browser for genes within 5 kb of the location provided by Illumina. Information on distance to the TSS or which region within a gene body the CpG is located was ascertained. Gene names were further updated using the NCBI Gene Database. To further understand functional enrichment, eFORGE (v2.0) was utilized [52].

## Supplementary information

**Additional file 1.**

## Data Availability

EAGeR data can be shared upon written request to the corresponding author. Data from the Generation R Study are available upon reasonable request to the director, Vincent Jaddoe (generation r@erasmusmc.nl), and subject to local rules and regulations. Due to ethical issues and consent, the PREDO datasets analyzed during the current study are not publicly available. However, an interested researcher can obtain a de-identified dataset after approval from the PREDO study board. Data requests may be subject to further review by the national register authority and by the ethical committees. Data can be obtained upon reasonable request from the PREDO study board (predo.study@helsinki. fi) or individual researchers.

## Abbreviations

CRP: C-reactive protein
hsCRP: High-sensitivity C-reactive protein
DNA: Deoxyribonucleic acid
EAGeR: Effects of Aspirin in Gestation and Reproduction
LDA: Low-dose aspirin
PACE: Pregnancy and Childhood Epigenetic
RBC: Red blood cell
