# Supplementary material for: Cord blood DNA methylation reflects cord blood C-reactive protein levels but not maternal levels: a longitudinal study and meta-analysis
Source: Clin Epigenetics. 2020 Apr 30;12:60. doi: 10.1186/s13148-020-00852-2 (PMC7193358; doi:10.1186/s13148-020-00852-2)
Supplement: Supplementary file 1 — Additional file 1. [file 13148_2020_852_MOESM1_ESM.zip › Inflamm and Methylation 04062020 additional file clean.docx]

**Supplemental Text**

Title: Pregnancy and delivery c-reactive protein concentrations and offspring DNA methylation

Normalization and QC steps

*INMA*: Cord blood was extracted using the Chemagen kit (Perkin Elmer). DNA concentration was determined by NanoDrop spectrophotometer (Thermo Scientific) and with the [Quant-iT PicoGreen dsDNA Assay Kit](https://www.lifetechnologies.com/order/catalog/product/P11496) (Life Technologies). Methylation data was produced in two different laboratories as part of two different projects: in the Genome Analysis Facility of the University Medical Center Groningen (UMCG) in Holland, and in the Bellvitge Biomedical Research Institute (IDIBELL, Barcelona). Both laboratories used the recommended Illumina protocol for the Infinium HumanMethylation450 beadchip. Briefly, 500 ng of DNA was bisulfite-converted using the EZ 96-DNA methylation kit following the manufacturer’s standard protocol, and DNA methylation measured using the Illumina Infinium HumanMethylation450 beadchip. DNA methylation data were preprocessed using the minfi package (Aryee et al. 2014). A series of steps were completed for quality control and data analysis. The first step was low quality sample removal. First, 2 samples with bad overall quality or with low detection p-value according to the output of the MethylAid package (van Iterson et al. 2014) were removed. Then, we removed 3 samples whose sex was wrongly predicted using shinyMethyl (Fortin, Fertig, and Hansen 2014). Following guidelines of Lehne^1^, we increased the stringency of the detection p-value threshold to 10-16 and we filtered 18 samples with a call rate lower than 98%. The second step was normalizing data with functional normalization. Correlation between SNP in replicates samples was checked and probes not measuring SNPs were discarded. 7,136 probes with a call rate lower than 95% were also removed. Probes in sexual chromosomes, crosshibridizing or containing SNPs were flagged but not removed at this point. ComBat was applied to remove batch effect (Johnson, Li, and Rabinovic 2007). Finally, duplicated samples were removed, prioritizing MeDALL samples over BREATHE samples. The final dataset consisted of 391 at age 0y and 476,946 probes. For the current study we used European ancestry children.

*Generation R:* DNA extracted (using the salting-out method) from blood samples taken at birth (cord blood collected by EDTA tube) was used for this analysis. 500 ng DNA per sample underwent bisulfite conversion using the EZ-96 DNA Methylation kit (Shallow) (Zymo Research Corporation, Irvine, USA). Samples were plated onto 96-well plates in no specific order. Samples were processed with the Illumina Infinium HumanMethylation450 BeadChip (Illumina Inc., San Diego, USA), which analyses methylation at 485,577 CpG sites. Preparation and normalization of the HumanMethylation450 BeadChip array data was performed according to the CPACOR workflow^1^ using the software package R^2^. In detail, the idat files were read using the minfi package. Probes that had a detection p-value above background (based on sum of methylated and unmethylated intensity values) ≥ 1E-16 were set to missing per array. Next, the intensity values were stratified by autosomal and non-autosomal probes and quantile normalized for each of the six probe type categories separately: type II red/green, type I methylated red/green and type I unmethylated red/green. Beta values were calculated as proportion of methylated intensity value on the sum of methylated+unmethylated+100 intensities. Arrays with observed technical problems such as failed bisulfite conversion, hybridization or extension, as well as arrays with a mismatch between sex of the proband and sex determined by the chr X and Y probe intensities were removed from subsequent analyses. Additionally, only arrays with a call rate > 95% per sample were processed further. The final dataset contained information on 469,242 CpGs for 1396 samples at birth. Methylation beta values outside the range of (25th percentile - 3*interquartile range, 75th percentile + 3*interquartile range) were set to missing.

*PREDO*: Cord blood samples were collected according to standard procedures. DNA was extracted at the National Institute for Health and Welfare, Helsinki, Finland and and the Finnish Institute of Molecular Medicine, University of Helsinki, Finland and methylation analyses were performed at the Max Planck Institute in Munich, Germany. DNA was bisulphite-converted using the EZ-96 DNA Methylation kit (Zymo Research, Irvine, CA). Samples were ran on Illumina 450K Methylation arrays and the arrays were scanned using the iScan System (Illumina Inc., San Diego, CA). The quality control pipeline was set up using the R-package minfi. 3 IDs were excluded as they were outliers in the median intensities. Furthermore, 20 IDs showed disconcordance between phenotypic sex and estimated sex and were excluded. Methylation beta-values were normalized using the funnorm function. We excluded any probes on chromosome X or Y, probes containing SNPs and cross-hybridizing probes. Furthermore, any CpGs with a detection p-value > 0.01 in at least 50% of the samples were excluded. The final dataset contains 428,619 CpGs and 834 IDs. We used ComBat to check and adjust for the batch effects (slide and well). Methylation beta values outside the range of (25th percentile - 3*interquartile range, 75th percentile + 3*interquartile range) were set to missing. Cord blood cell counts were estimated for seven cell types (nucleated red blood cells, granulocytes, monocytes, natural killer cells, B cells, CD4(+)T cells, and CD8(+)T cells) using the method of Bakulski et al. which is incorporated in the R-package minfi.

CRP assay methods

*INMA*: Maternal blood was collected at recruitment (mean ± SD, 13.4 ± 1.7 weeks of gestation). Serum CRP values were determined by turbidimetric assay using a Hitachi modular analyzer system (Roche Modular DPP, Hitachi Ltd, Tokyo, Japan) at the Laboratori de Referencia de Catalunya. The minimum detectable concentration of CRP was 0.2 mg/dL. For participants who had a CRP value below the detection limit (15.9%), we imputed a value of half of the detection limit.

*Generation R****:*** Maternal non-fasting venous blood samples were collected in EDTA tube in early pregnancy and transported to the regional laboratory (Star-MDC, Rotterdam, The Netherlands) for processing and storage^3^. Blood samples were stored at −80°C. High-sensitivity CRP concentrations were measured in EDTA plasma samples at the Department of Clinical Chemistry of the Erasmus MC. CRP levels were analyzed using an immunoturbidimetric assay on the Architect System (Abbot Diagnostics B.V., Hoofddorp, The Netherlands). The within run precision for CRP was 1.3% at 12.9 mg/L and 1.2% at 39.9 mg/L. The lowest level of detection was 0.2 mg/L.

*PREDO*: The participants came for venous blood sampling between 7-9 AM, after having been instructed to fast for a minimum of 10 hours. The samples were drawn from an antecubital vein, and the plasma was separated immediately. EDTA plasma samples were stored at -80°C until analyzed. The concentration of hsCRP (mg/L) in plasma was analyzed with a highly sensitive Olympus CRP immunoturbidometric assay and Olympus AU680 analyzer (Beckman Coulter Inc., CA, USA). The functional sensitivity of the hsCRP method was 0.15 mg/L.

Covariate information

*INMA:*  Information on maternal age was collected by questionnaire at enrolment (week 12 of pregnancy). Maternal age was used as a continuous covariate. Pregnant women were asked about maternal smoking at week 32 of pregnancy. The variable has been divided in three categories; no smoking, smoking throughout the pregnancy and smoking, but stopped early in pregnancy. (in the table we only show the results of the group in which pregnant women smoked throughout the pregnancy). Parental socioeconomic status was based on parental education level assessed by maternal questionnaire at week 12 and classified as low (primary or less and secondary) and high (university) separately from maternal and paternal. Maternal height was measured, and maternal pre-pregnancy weight was reported by the mother during the first prenatal visit. Gestational age at blood sampling was calculated based on last menstrual period (LMP) reported at recruitment and confirmed using estimates based on ultrasound examina­tion in the 12th week of gestation. When the difference between the LMP reported at recruitment and estimated from the ultra­sound was ≥ 7 days (n = 91; 16%), we esti­mated LMP using a quadratic regression formula defined by Westerway et al.^2^ Birth weight was obtained from clinical records.

*Generation R*: Information on maternal age and educational level were collected by questionnaire at enrolment. Maternal socio-economic status was based on maternal educational level, and classified as low/middle (primary or less and secondary) or high (university). Maternal height and weight were measured at enrolment, and BMI was subsequently calculated. Information on maternal smoking was obtained by repeated questionnaires during pregnancy, and was classified as no smoking, smoking until pregnancy was known, and continued smoking.

*PREDO*: In the models, we adjusted for maternal age at childbirth, maternal first trimester BMI measured at first antenatal clinic visit, maternal smoking during pregnancy, maternal educational level (basic, secondary, tertiary), and gestational age at birth. These data were derived from the Finnish Medical Birth Register (MBR), HILMO or medical records. In addition, we adjusted for population stratification with four first multidimensional scaling components derived from the genome-wide genotyping data and estimated cell type composition.

Biological functions of genes located at/near the CpGs associated with newborn CRP levels

The strongest association (15% methylation difference) for newborn CRP was with DNA methylation at cg13138089 which is located 16bp after *ECEL1P2* (endothelin converting enzyme like 1 pseudogene 2) and 4kb after *PLAP* (placental alkaline phosphatase). Thus, it is unclear exactly which gene methylation at this site may impact. Methylation of *ECEL1P2* has been linked with smoking^3^ and may play a potential role in age-related development.^4^ Methylation of the exon of *SPEG* (striated muscle preferentially expressed protein kinase) increased with CRP. While the protein is related to myocyte cytoskeletal and cardiac development^5^, methylation has not been examined. *ARHGEF17* (Rho guanine nucleotide exchange factor (GEF) 17) is expressed highly in artery tissue and rare genetic variants associated with intracranial aneurysms.^6^ Genetic variants of OLFML2A (olfactomedin-like 2A) are also associated with intracranial aneurysms in a separate study and the class of olfactomedin proteins plays a role in cellular differentiation and neurogenesis.^7,8^ cg13558754 is in the intron of *HSPB6* (heat shock protein, alpha-crystallin-related, B6), also known as HSP20, has vascular properties by promoting angiogenesis and is cardioprotective.^9^ It also has been found to be protective of development of several types of cancer, with hypermethylation found in tumor samples.^10^ A CpG located in the promoter region of another heat shock protein gene (*HSPE1*, heat shock 10kDa protein 1 (chaperonin 10)) was also associated with higher methylation. Heat shock proteins (HSPs) as a class, respond to cellular stress, chaperoning other proteins and activating immune responses by stimulating antigen-presenting cells such as macrophages and leading to inflammatory response.^11^ cg17990365 located in the exon of *IFITM3* (interferon induced transmembrane protein 3), plays a role in protection against viruses, with recent findings in a knockout mouse model that it may be specifically cardioprotective in response to viral infection by secondarily dampening inflammatory cytokines to prevent tissue damage.^12^ Methylation and overexpression of *ESRP2* (epithelial splicing regulatory protein 2) has been associated with breast cancer prognosis^13^ through its role in posttranscriptional gene regulation of extracellular matrix transcripts by binding them to either lead to exon skipping or exon inclusion and thus producing isoforms with different functions.^14^ *DIRC3* (disrupted in renal carcinoma 3) has been associated with thyroid cancer in several GWAS.^15^ Its function and epigenetic regulation, however, are unknown. Cg24340661 is located in the exon of *ILDR2* (immunoglobulin-like domain containing receptor 2) and upstream of *MAEL* (maelstrom spermatogenic transposon silencer). Both genes are expressed in the testis. *ILDR2* has immunological function.^16^ cg23200634 is located <2kb after *IGHMBP2* (immunoglobulin mu binding protein 2) and its methylation associated with dementia.^17^ *TEPP* (testis, prostate and placenta expressed, transcript variant 2) is relatively unknown. *BSX* (brain specific homeobox) is involved in energy balance, with the hormones ghrelin and leptin capable of altering its expression in the arcuate nucleus.^18^ *DMTN* (dematin actin binding protein) helps with maintaining the red cell membrane, important for erythrocytes to pass through small vessels.^19,20^ SNPs of *ZADH2* (zinc binding alcohol dehydrogenase domain containing 2) has been associated with angiogenic factor vascular endothelial growth factor (VEGF) levels,^21^ which has been correlated with circulating CRP. *ZADH2* methylation in blood was found to be hypomethylated in individuals with Alzheimer’s disease.^22^ *DEPDC1* (DEP domain containing 1) is associated with cell proliferation and angiogenesis through induction of chemokines in the inflammation pathway.^23^ *TRIM6* (tripartite motif containing 6) has been associated with exacerbating cardiac injury in a mouse model and suspected to be activated by hypoxia and inflammatory factors.^24^ *UNC79* (unc-79 homolog) codes for a protein that is part of a cation channel (NALCN) regulating sodium leak and thus mutations documented to a range of neurodevelopment disorders.^25^ Serine peptidase inhibitors (SERPIN) play a role in cellular processes including inflammation and its mutations associated with development of allergies and its sequalae.^26,27^ SERPINB10 (serpin peptidase inhibitor, clade B (ovalbumin), member 10) specifically has been found to play a role in asthma by increasing eosinophilic airway inflammation.^27^ Not much is known about *ANKRD60* (ankyrin repeat domain 60) although it was associated with height among Koreans in a GWAS.^28^ Much has been published regarding *JMJD1C* (jumonji domain containing 1C) polymorphisms in association with leukemia^29^, platelet aggregation, sex steroid hormone control^30^, and infertility.^31^ Functionally, it has been shown to demethylate a mediator of DNA-damage checkpoint 1 (*MDMDC1*) which plays a role in repairing double stranded breaks.^32^ It has also been associated with VEGF levels in the aforementioned GWAS study that found associations with *ZADH2*.^21^ *RGS5* (regulator of G-protein signaling 5) produces signal molecules involved in response to hypoxia and mediates effects of VEGF.^33^ Its polymorphisms are associated with coronary artery disease^34^ and hypertension.^35^ Cg21542650 was located >500bp before *FKBP4* (FK506 binding protein 4). FK506 is one of the co-chaperone proteins involved in binding to the androgen receptor and part of the final complex necessary for hormone binding.^36^ It has been examined as part of understanding androgen dysregulation in polycystic ovarian syndrome (PCOS)^36^ and its methylation with breast cancer.^37^

**Supplemental Table 1. Study descriptive characteristics**

|  | INMA | Gen R | PREDO |
| --- | --- | --- | --- |
| N | 266 | 773 | 242 |
| Smoking during pregnancy, N (%) | 38 (14.3) | 169 (21.8) | 14 (5.8) |
| Female, N (%) | 133 (50) | 393 (51) | 122 (50.4) |
| Education mother, high | 82 (31) (University level) | 549 (71.0) | 76 (31.4) |
| Education father, high | 47 (18) (University level) | n/a | n/a |
| Maternal pre/early-pregnancy BMI, mean (SD) | 23.8 (4.1) | 23.4 (3.2) | 26.0 (6.0) |
| Gestational age, mean (SD) | 39.9 (1.4) | 40.2 (1.5) | 39.8 (1.7) |
| Birthweight, mean (SD) | 3260 (430) | 3550 (494) | 3506 (542) |

**Supplemental Table 2. Genomic Inflation of first trimester models**

| *INMA* | **Sample size** | **Lambda** |
| --- | --- | --- |
| Model 1 | 266 | 0.8775 |
| Model 2: Balkulski cell type adjusted | 266 | 0.9282 |
| Model 3: Gervin, Salas cell type adjusted | 266 | 1.1494 |
| *Generation R* | **Sample size** | **Lambda** |
| Model 1 | 773 | 1.307 |
| Model 2: Balkulski cell type adjusted | 773 | 1.066 |
| Model 3: Gervin, Salas cell type adjusted | 773 | 1.127 |
| PREDO | **Sample size** | **Lambda** |
| Model 1 | 242 | 1.0316 |
| Model 2: Balkulski cell type adjusted | 242 | 0.8328 |
| Model 3: Gervin, Salas cell type adjusted | n/a | n/a |
| EAGeR | **Sample size** | **Lambda** |
| Model 1 | 322 | 0.8865 |
| Model 2: Balkulski cell type adjusted | 322 | 0.9627 |
| Model 3: Gervin, Salas cell type adjusted | 322 | 0.9474 |

All models adjusted for maternal age, race (as applicable), socioeconomic income (by education, income or other cohort specific factors), maternal BMI and smoking)

**Supplemental Table 3. Differences in newborn DNA methylation when comparing mothers in the top tertile of CRP over all trimesters of pregnancy (n=61) and mothers in the lowest tertile (n=59)***

| probeID | BETA | SE | P_VAL | p.FDR | chr | pos | Gene | Relation_to_Island |
| --- | --- | --- | --- | --- | --- | --- | --- | --- |
| cg08964730 | -0.05058 | 0.009083 | 2.58E-08 | 0.021 | 6 | 32634009 | HLA-DQB1 | S_Shore |
| cg08230969 | -0.07872 | 0.01472 | 8.86E-08 | 0.027 | 3 | 101755465 |  | OpenSea |
| cg13418576 | 0.05276 | 0.009898 | 9.80E-08 | 0.027 | 9 | 126105738 |  | S_Shelf |
| ch.9.2793850R | 0.01216 | 0.00231 | 1.40E-07 | 0.028 | 9 | 2803850 | PUM3 | OpenSea |
| cg24524879 | -0.02112 | 0.004062 | 2.00E-07 | 0.030 | 5 | 38148555 | LINC02119 | OpenSea |
| cg22111043 | 0.01052 | 0.002031 | 2.20E-07 | 0.030 | 7 | 45019005 | MYO1G | OpenSea |

*Model 3: adjusted for maternal age, race (as applicable), socioeconomic income (by education, income or other cohort specific factors), maternal BMI and smoking) and cell type distribution (Gervin, Salas et al.)

**Supplemental Table 4. Transcription factor enrichment corresponding to the 33 CpGs identified as significant for CRP at delivery and newborn methylation**

| Transcription Factor | Database | p-value | q-value | Notes |
| --- | --- | --- | --- | --- |
| NFATC1_NFAT_1 | Taipale/SELEX | 6.66E-05 | 0.003591 | Tcells, CRP is one of the targets during interactions |
| Atf4.mouse_bZIP_1 | Taipale/SELEX | 3.11E-05 | 0.003591 | widely expressed in mammalian cells among others in all the immune cell lineages |
| ATF4_bZIP_1 | Taipale/SELEX | 3.2E-05 | 0.003591 | widely expressed in mammalian cells among others in all the immune cell lineages |
| TBX20_TBX_2 | Taipale/SELEX | 6.62E-05 | 0.003591 | T-box family involved in circulatory system developmentprotein-protein interactions with CRP |
| V_CEBPDELTA_Q6 | TRANSFAC | 6.58E-05 | 0.003591 | hypoxia pathway |
| Nr2f2_primary | UniProbe | 9.25E-05 | 0.004154 | steroid related no direct CRP |
| Rxra_secondary | UniProbe | 0.000153 | 0.00458 | steroid and thyroid receptors, related to cyclins and calmodulins |
| Sox7_secondary | UniProbe | 0.000147 | 0.00458 | regulation of embryonic development |
| Rara_primary | UniProbe | 0.00014 | 0.00458 | retinoic acid receptors |
| TFAP2C_TFAP_5 | Taipale/SELEX | 0.000198 | 0.005335 | retinoic acid receptors |
| V_ERR2_01 | TRANSFAC | 0.00023 | 0.005632 | several G proteins and transporters |
| Esrra_primary | UniProbe | 0.000282 | 0.006307 | thryoid receptor and estrogen receptors |
| V_NFMUE1_Q6 | TRANSFAC | 0.000304 | 0.006307 | unknown |
| V_YY1_Q6_02 | TRANSFAC | 0.00034 | 0.006538 | repressor of multiple promoters |
| V_HSF1_Q6 | TRANSFAC | 0.000365 | 0.006563 | heat shock proteins |
| V_ATF1_Q6 | TRANSFAC | 0.000579 | 0.009748 | threonine/serine kinases |
| MA0018.1-CREB1 | JASPAR | 0.001366 | 0.020458 | leucine zipper |
| V_STAT1_05 | TRANSFAC | 0.001363 | 0.020458 | activation for several cytokines |
| V_STAT3_03 | TRANSFAC | 0.001891 | 0.026821 | cell growth and apoptosis |
| V_PUR1_Q4 | TRANSFAC | 0.002038 | 0.027456 | unknown |
| MA0149.1-EWSR1-FLI1 | JASPAR | 0.002359 | 0.028891 | Ewing sarcoma target |
| V_DAX1_01 | TRANSFAC | 0.002304 | 0.028891 | unknown |
| V_STAF_01 | TRANSFAC | 0.002712 | 0.031776 | zinc finger |
| MA0088.1-znf143 | JASPAR | 0.003105 | 0.033471 | zinc finger |
| TFAP2C_TFAP_3 | Taipale/SELEX | 0.003051 | 0.033471 | retinoic acid receptors |
| V_STAF_02 | TRANSFAC | 0.003687 | 0.038214 | zinc finger |
| TFAP2A_AP2_6 | Taipale/SELEX | 0.003936 | 0.039283 | BOFS syndrome |
| V_AP4_01 | TRANSFAC | 0.004402 | 0.042365 | adaptor complexes |
| Tcfap2a.mouse_TFAP_3 | Taipale/SELEX | 0.004669 | 0.04241 | retinoic acid receptors |
| NRF1_NRF_1 | Taipale/SELEX | 0.004879 | 0.04241 | genes for cell respiration |
| V_NRF1_Q6 | TRANSFAC | 0.004869 | 0.04241 | genes for cell respiration |

Additional supplemental results are publicly available in online repository (hosted by NIH Figshare):

- Supplemental Table A1: EAGeR Model 1 First Trimester (week 8) CRP
- Supplemental Table A2: EAGeR Model 2 First Trimester (week 8) CRP
- Supplemental Table A3: EAGeR Model 3 First Trimester (week 8) CRP
- Supplemental Table A4: EAGeR Model 3 First Trimester (sensitivity analysis) CRP
- Supplemental Table B1: EAGeR Model 1 Second Trimester (week 20) CRP
- Supplemental Table B2: EAGeR Model 2 Second Trimester (week 20) CRP
- Supplemental Table B3: EAGeR Model 3 Second Trimester (week 20) CRP
- Supplemental Table B4: EAGeR Model 3 Second Trimester (sensitivity analysis) CRP
- Supplemental Table C1: EAGeR Model 1 Third Trimester (week 36) CRP
- Supplemental Table C2: EAGeR Model 2 Third Trimester (week 36) CRP
- Supplemental Table C3: EAGeR Model 3 Third Trimester (week 36) CRP
- Supplemental Table C4: EAGeR Model 3 Third Trimester (sensitivity analysis) CRP
- Supplemental Table D1: EAGeR Model 1 Cumulative (AUC) CRP
- Supplemental Table D2: EAGeR Model 2 Cumulative (AUC) CRP
- Supplemental Table D3: EAGeR Model 3 Cumulative (AUC) CRP
- Supplemental Table E1: EAGeR Model 1 Persistent (extreme tertiles) CRP
- Supplemental Table E2: EAGeR Model 2 Persistent (extreme tertiles) CRP
- Supplemental Table E3: EAGeR Model 3 Persistent (extreme tertiles) CRP
- Supplemental Table F1: EAGeR Model 1 Cord Blood (delivery) CRP
- Supplemental Table F2: EAGeR Model 2 Cord Blood (delivery) CRP
- Supplemental Table F3: EAGeR Model 3 Cord Blood (delivery) CRP

**Supplemental Figure Legends**

Supplemental Figure 1. Manhattan plot of results for randomization to low dose aspirin in EAGeR Model adjusted for maternal age, smoking status, income, pre-pregnancy BMI, plate and cell count distribution (as estimated in Gervin et al.^38^). Line indicates Bonferroni significance at p=6x10^-8^.

Supplemental Figure 2. Differences in results by cell type adjustment in newborn CRP and methylation models

Supplemental Figure 3. Transcription factors identified from eFORGE

Supplemental Figure 4. Summary of findings on pregnancy and delivery CRP and newborn DNA methylation

References

1. Lehne B, Drong AW, Loh M, et al. A coherent approach for analysis of the Illumina HumanMethylation450 BeadChip improves data quality and performance in epigenome-wide association studies. *Genome Biol.* 2015;16:37.

2. Westerway SC, Davison A, Cowell S. Ultrasonic fetal measurements: new Australian standards for the new millennium. *Aust N Z J Obstet Gynaecol.* 2000;40(3):297-302.

3. Ambatipudi S, Cuenin C, Hernandez-Vargas H, et al. Tobacco smoking-associated genome-wide DNA methylation changes in the EPIC study. *Epigenomics.* 2016;8(5):599-618.

4. Salpea P, Russanova VR, Hirai TH, et al. Postnatal development- and age-related changes in DNA-methylation patterns in the human genome. *Nucleic Acids Res.* 2012;40(14):6477-6494.

5. Quick AP, Wang Q, Philippen LE, et al. SPEG (Striated Muscle Preferentially Expressed Protein Kinase) Is Essential for Cardiac Function by Regulating Junctional Membrane Complex Activity. *Circ Res.* 2017;120(1):110-119.

6. Yang X, Li J, Fang Y, et al. Rho Guanine Nucleotide Exchange Factor ARHGEF17 Is a Risk Gene for Intracranial Aneurysms. *Circ Genom Precis Med.* 2018;11(7):e002099.

7. Hong EP, Kim BJ, Cho SS, et al. Genomic Variations in Susceptibility to Intracranial Aneurysm in the Korean Population. *J Clin Med.* 2019;8(2).

8. Anholt RR. Olfactomedin proteins: central players in development and disease. *Front Cell Dev Biol.* 2014;2:6.

9. Zhang X, Wang X, Zhu H, et al. Hsp20 functions as a novel cardiokine in promoting angiogenesis via activation of VEGFR2. *PLoS One.* 2012;7(3):e32765.

10. Wang J, Duan Y, Meng QH, et al. Integrated analysis of DNA methylation profiling and gene expression profiling identifies novel markers in lung cancer in Xuanwei, China. *PLoS One.* 2018;13(10):e0203155.

11. Martine P, Rebe C. Heat Shock Proteins and Inflammasomes. *Int J Mol Sci.* 2019;20(18).

12. Kenney AD, McMichael TM, Imas A, et al. IFITM3 protects the heart during influenza virus infection. *Proceedings of the National Academy of Sciences of the United States of America.* 2019;116(37):18607-18612.

13. Heilmann K, Toth R, Bossmann C, Klimo K, Plass C, Gerhauser C. Genome-wide screen for differentially methylated long noncoding RNAs identifies Esrp2 and lncRNA Esrp2-as regulated by enhancer DNA methylation with prognostic relevance for human breast cancer. *Oncogene.* 2017;36(46):6446-6461.

14. De Craene B, Berx G. Regulatory networks defining EMT during cancer initiation and progression. *Nat Rev Cancer.* 2013;13(2):97-110.

15. Son HY, Hwangbo Y, Yoo SK, et al. Genome-wide association and expression quantitative trait loci studies identify multiple susceptibility loci for thyroid cancer. *Nat Commun.* 2017;8:15966.

16. Hecht I, Toporik A, Podojil JR, et al. ILDR2 Is a Novel B7-like Protein That Negatively Regulates T Cell Responses. *Journal of immunology.* 2018;200(6):2025-2037.

17. Taskesen E, Mishra A, van der Sluis S, et al. Susceptible genes and disease mechanisms identified in frontotemporal dementia and frontotemporal dementia with Amyotrophic Lateral Sclerosis by DNA-methylation and GWAS. *Sci Rep.* 2017;7(1):8899.

18. Kaji T, Nonogaki K. Role of homeobox genes in the hypothalamic development and energy balance. *Front Biosci (Landmark Ed).* 2013;18:740-747.

19. Zail S. Clinical disorders of the red cell membrane skeleton. *Crit Rev Oncol Hematol.* 1986;5(4):397-453.

20. Khanna R, Chang SH, Andrabi S, et al. Headpiece domain of dematin is required for the stability of the erythrocyte membrane. *Proceedings of the National Academy of Sciences of the United States of America.* 2002;99(10):6637-6642.

21. Choi SH, Ruggiero D, Sorice R, et al. Six Novel Loci Associated with Circulating VEGF Levels Identified by a Meta-analysis of Genome-Wide Association Studies. *PLoS Genet.* 2016;12(2):e1005874.

22. Madrid A, Hogan KJ, Papale LA, et al. DNA Hypomethylation in Blood Links B3GALT4 and ZADH2 to Alzheimer's Disease. *J Alzheimers Dis.* 2018;66(3):927-934.

23. Guo W, Li H, Liu H, Ma X, Yang S, Wang Z. DEPDC1 drives hepatocellular carcinoma cell proliferation, invasion and angiogenesis by regulating the CCL20/CCR6 signaling pathway. *Oncol Rep.* 2019;42(3):1075-1089.

24. Zeng G, Lian C, Yang P, Zheng M, Ren H, Wang H. E3-ubiquitin ligase TRIM6 aggravates myocardial ischemia/reperfusion injury via promoting STAT1-dependent cardiomyocyte apoptosis. *Aging (Albany NY).* 2019;11(11):3536-3550.

25. Bramswig NC, Bertoli-Avella AM, Albrecht B, et al. Genetic variants in components of the NALCN-UNC80-UNC79 ion channel complex cause a broad clinical phenotype (NALCN channelopathies). *Human genetics.* 2018;137(9):753-768.

26. Marenholz I, Grosche S, Kalb B, et al. Genome-wide association study identifies the SERPINB gene cluster as a susceptibility locus for food allergy. *Nat Commun.* 2017;8(1):1056.

27. Mo Y, Zhang K, Feng Y, et al. Epithelial SERPINB10, a novel marker of airway eosinophilia in asthma, contributes to allergic airway inflammation. *Am J Physiol Lung Cell Mol Physiol.* 2019;316(1):L245-L254.

28. Kim JJ, Lee HI, Park T, et al. Identification of 15 loci influencing height in a Korean population. *J Hum Genet.* 2010;55(1):27-31.

29. Izaguirre-Carbonell J, Christiansen L, Burns R, et al. Critical role of Jumonji domain of JMJD1C in MLL-rearranged leukemia. *Blood Adv.* 2019;3(9):1499-1511.

30. Grigorova M, Punab M, Poolamets O, Adler M, Vihljajev V, Laan M. Genetics of Sex Hormone-Binding Globulin and Testosterone Levels in Fertile and Infertile Men of Reproductive Age. *J Endocr Soc.* 2017;1(6):560-576.

31. Sujit KM, Sarkar S, Singh V, et al. Genome-wide differential methylation analyses identifies methylation signatures of male infertility. *Hum Reprod.* 2018;33(12):2256-2267.

32. Watanabe S, Watanabe K, Akimov V, et al. JMJD1C demethylates MDC1 to regulate the RNF8 and BRCA1-mediated chromatin response to DNA breaks. *Nat Struct Mol Biol.* 2013;20(12):1425-1433.

33. Jin Y, An X, Ye Z, Cully B, Wu J, Li J. RGS5, a hypoxia-inducible apoptotic stimulator in endothelial cells. *J Biol Chem.* 2009;284(35):23436-23443.

34. Li Y, Yan H, Guo J, et al. Down-regulated RGS5 by genetic variants impairs endothelial cell function and contributes to coronary artery disease. *Cardiovasc Res.* 2019.

35. Xiao B, Zhang Y, Niu WQ, Gao PJ, Zhu DL. Haplotype-based association of regulator of G-protein signaling 5 gene polymorphisms with essential hypertension and metabolic parameters in Chinese. *Clinical chemistry and laboratory medicine : CCLM / FESCC.* 2009;47(12):1483-1488.

36. Ketefian A, Jones MR, Krauss RM, et al. Association study of androgen signaling pathway genes in polycystic ovary syndrome. *Fertil Steril.* 2016;105(2):467-473 e464.

37. Guerrero-Preston R, Hadar T, Ostrow KL, et al. Differential promoter methylation of kinesin family member 1a in plasma is associated with breast cancer and DNA repair capacity. *Oncol Rep.* 2014;32(2):505-512.

38. Gervin K, Salas LA, Bakulski KM, et al. Systematic evaluation and validation of reference and library selection methods for deconvolution of cord blood DNA methylation data. *Clinical epigenetics.* 2019;11(1):125.
