# Supplementary material for: Cord blood DNA methylation reflects cord blood C-reactive protein levels but not maternal levels: a longitudinal study and meta-analysis
Source: Clin Epigenetics. 2020 Apr 30;12:60. doi: 10.1186/s13148-020-00852-2 (PMC7193358; doi:10.1186/s13148-020-00852-2)

Supplemental Figure 4: Summary of findings on pregnancy and delivery CRP and newborn DNA methylation

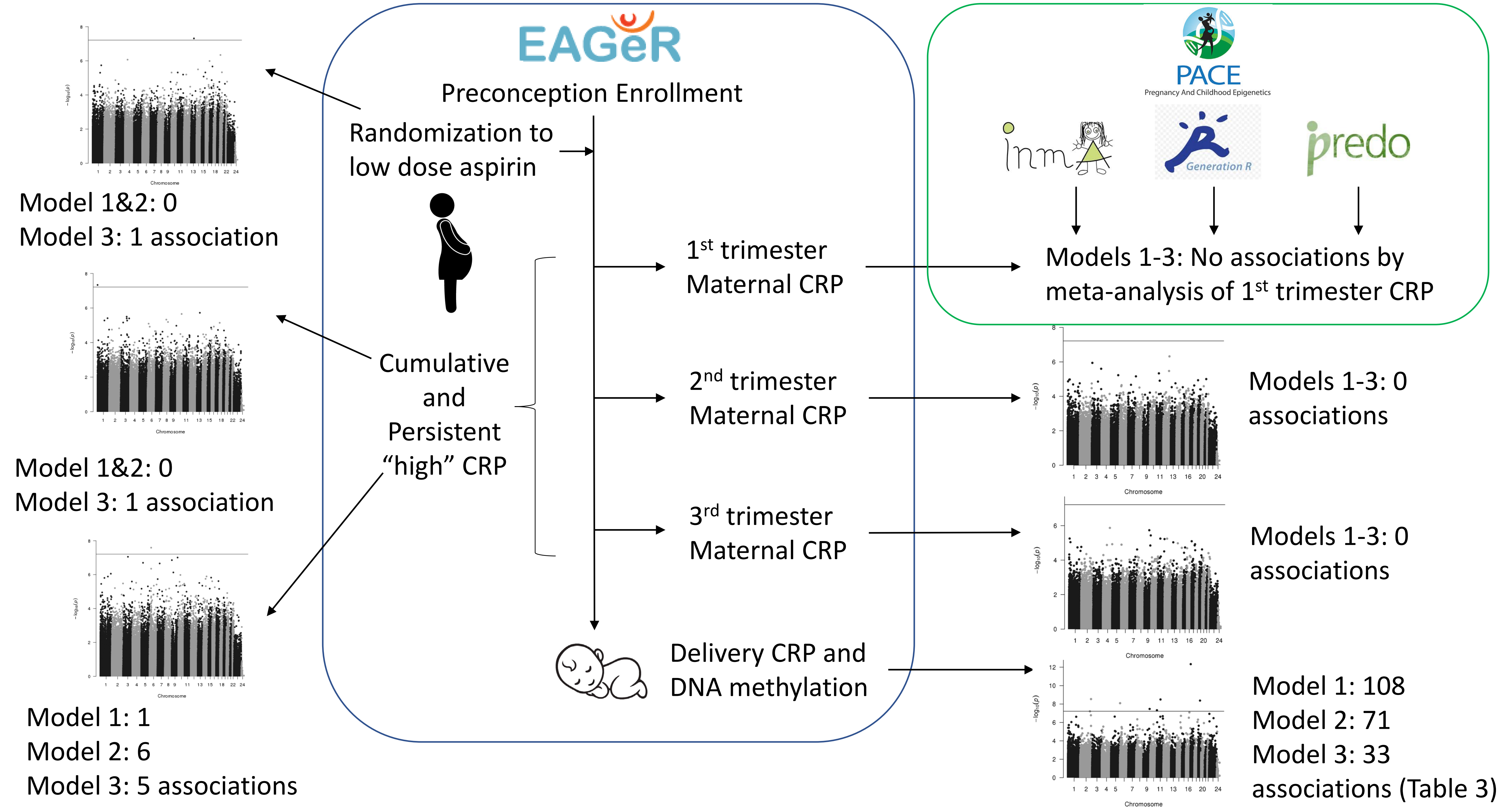

Supplement: Supplementary file 1 — Additional file 1. [file 13148_2020_852_MOESM1_ESM.zip › SupplFig4_Study_Summary_Diagram_02202020.pdf]
