# Supplementary figures and images for: Cord blood DNA methylation reflects cord blood C-reactive protein levels but not maternal levels: a longitudinal study and meta-analysis
Source: Clin Epigenetics. 2020 Apr 30;12:60. doi: 10.1186/s13148-020-00852-2 (PMC7193358; doi:10.1186/s13148-020-00852-2)

Crude (108 hits)

Model 1 (71 hits)

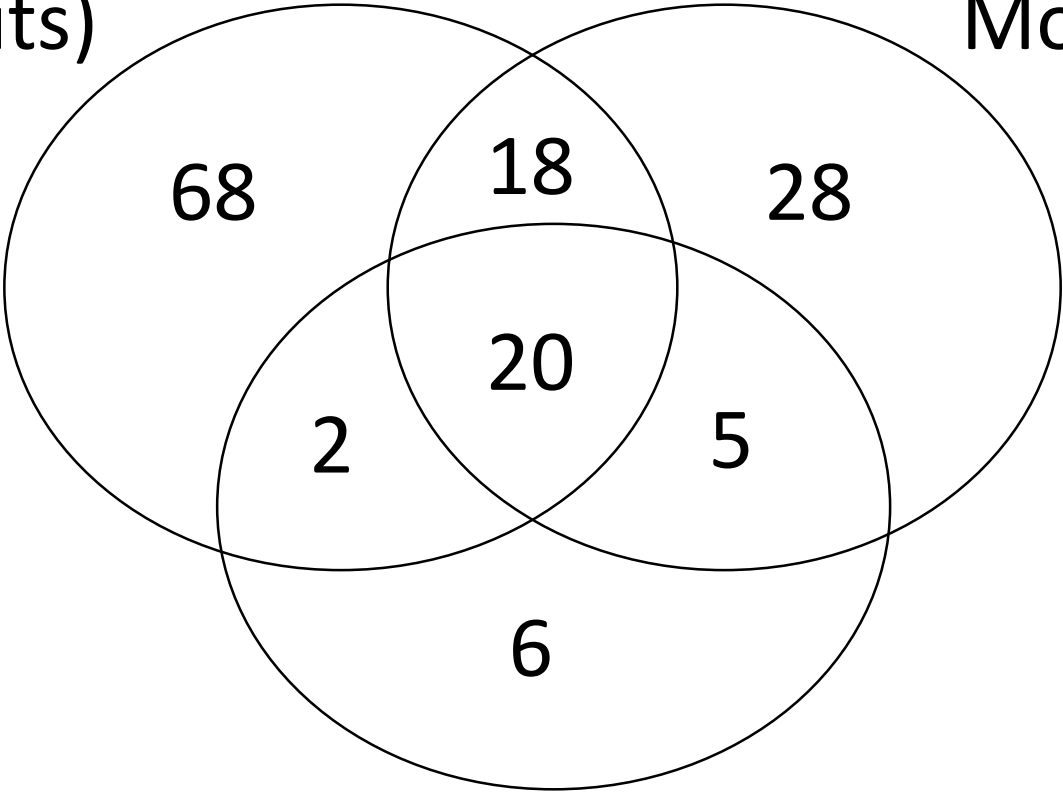

Model 2 (33 hits)

Supplement: Supplementary file 1 — Additional file 1. [file 13148_2020_852_MOESM1_ESM.zip › Supplemental_Fig2_Venn_diagram.pdf]

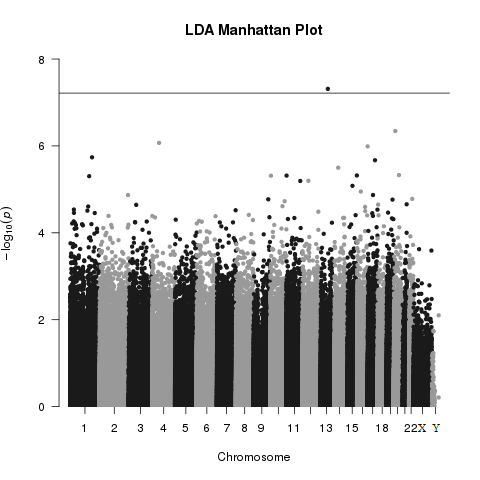

Supplement: Supplementary file 1 — Additional file 1. [file 13148_2020_852_MOESM1_ESM.zip › Supplemental_Figure1.png]

# Probeset TF Associations

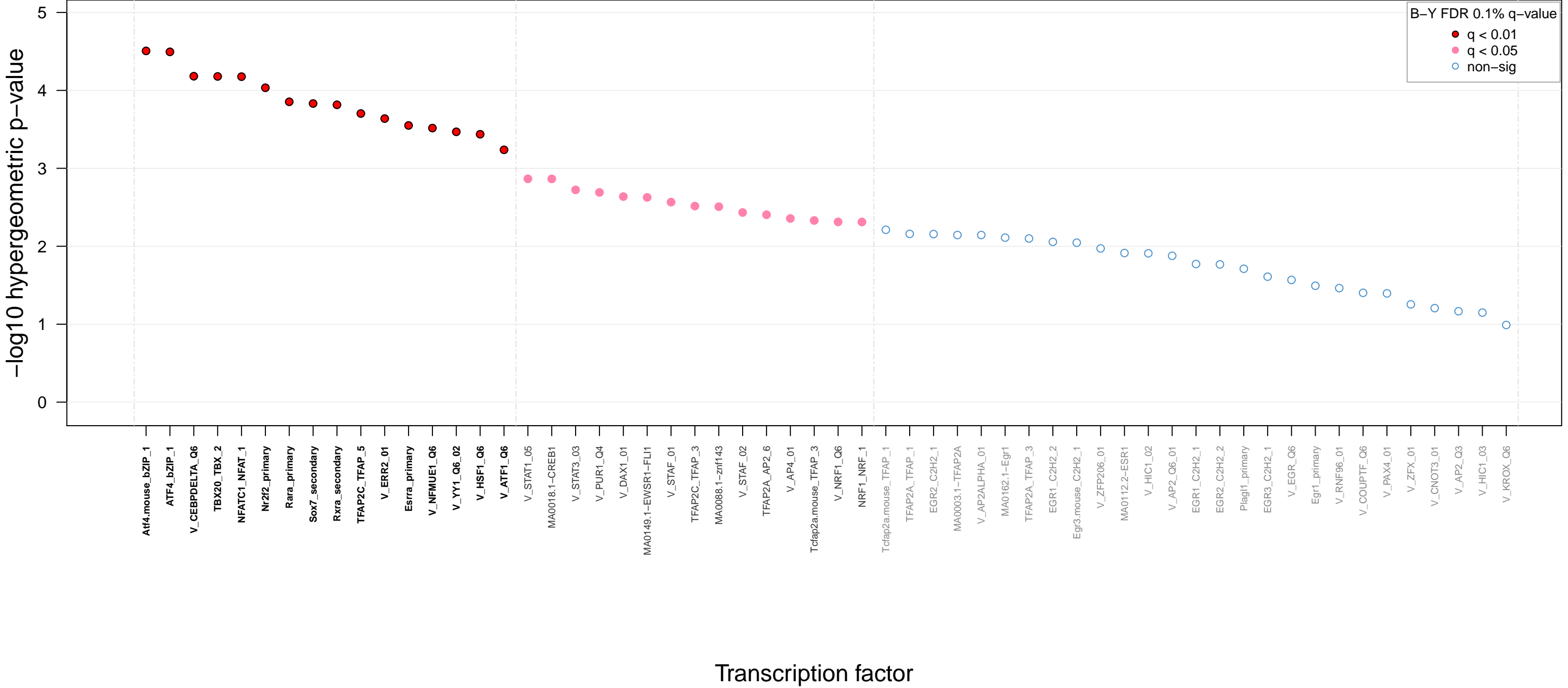

Supplement: Supplementary file 1 — Additional file 1. [file 13148_2020_852_MOESM1_ESM.zip › Supplemental_Figure3_eFORGE-TF.2019-11-02.pdf]
